# Supplementary material for: Eating Speed Is Associated with the Presence of Sarcopenia in Older Patients with Type 2 Diabetes: A Cross-Sectional Study of the KAMOGAWA-DM Cohort
Source: Nutrients. 2022 Feb 11;14(4):759. doi: 10.3390/nu14040759 (PMC8878855; doi:10.3390/nu14040759)
Supplement: Supplementary file 1 [file nutrients-14-00759-s001.zip › nutrients-1584463-supplementary.pdf]

Supplementary Materials

ID __________ Date of entry ________

1. When were you diagnosed as having diabetes?

______ years or __ For the first time

2. Please show height and body weight.

____ cm ____ kg

3. What was your weight when you were 20 years old and what is your maximum weight so far and how old were you at that time?

20 year old ____kg Maximum body weight ____ kg and _____ years old

4. Do you have some relatives who had diabetes?

__ None __ Father __ Mother __ The others

5. Regarding smoking: please check one of the boxes.

__ Current smoker __ Past smoker __Never smoker

If check current smoker or past smoker, how many cigarettes per day have you smoking (or smoked) for how many years?

_____ / day and ____ years

6. Regarding drinking. Do you drink alcohol? Please check one of the boxes.

__ Every day or almost every day __ 3 to 4 times per week __ 1 to 2 times per week

__ Less than once a week __ None

7. Regarding exercise. Do you exercise regularly? Please check one of the boxes.

__ Yes __ No

If yes, what kind of exercise do you do and for how long?

What kind ________

How long ________hour per __month __week __day
